# The Prevalence of Supernumerary Teeth in a Sample of Non-Syndromic Young Patients from Greece

**DOI:** 10.3390/dj13070317

**Published:** 2025-07-14

**Authors:** Nefeli Katanaki, Miltiadis A. Makrygiannakis, Eleftherios G. Kaklamanos

**Affiliations:** 1 Private Practice, 81132 Mytilini, Lesvos, Greece; 2 School of Dentistry, National and Kapodistrian University of Athens, 11527 Athens, Greece; 3 School of Dentistry, European University Cyprus, 2404 Nicosia, Cyprus; 4 School of Dentistry, Aristotle University of Thessaloniki, 54124 Thessaloniki, Greece; 5 Hamdan Bin Mohammed College of Dental Medicine, Mohammed Bin Rashid University of Medicine and Health Sciences, Dubai P.O. Box 505055, United Arab Emirates

**Keywords:** supernumerary teeth, hyperdontia, prevalence, Lesvos, Greece

## Abstract

**Background/Objectives**: Supernumerary teeth, or hyperdontia, refer to a developmental anomaly defined by the presence of additional teeth beyond the normal dentition. Hyperdontia may result in clinical complications including delayed eruption, crowding, and malocclusion. Despite its prevalence having been studied in various populations, data from geographically isolated or peripheral groups remain limited. This study aimed to investigate the prevalence and distribution of supernumerary teeth in a sample of children and adolescents from the island of Lesvos, Greece. **Methods**: A retrospective cross-sectional study was conducted using panoramic radiographs from 621 Caucasian children aged 9–16 years who attended orthodontic or general/pediatric dental clinics in Mytilini, Lesvos island, Greece. Radiographs were examined for the presence, number, type, and location of supernumerary teeth. The analysis included data to explore gender and arch distribution. **Results**: Supernumerary teeth were identified in 15 individuals, corresponding to a prevalence of 2.4%. A slightly higher occurrence was observed in males (1.4%) than in females (1%). The majority of supernumerary teeth were situated in the maxillary arch (1.9%). Mesiodens represented the most frequently observed type, followed by supernumerary lateral incisors, paramolars, and a single supernumerary central incisor. **Conclusions**: The prevalence of supernumerary teeth in this population is consistent with reported findings. Mesiodens was the most frequently observed type, with a predominance in the maxillary arch. Early detection of supernumerary teeth is crucial for accurate diagnosis and effective management.

## 1. Introduction

Hyperdontia, also known as supernumerary teeth, is a condition that arises during the dental lamina formation stage and encompasses the induction and proliferation phases [1]. It most frequently affects the maxillary anterior teeth, especially the anterior area, namely the central incisors, followed by maxillary and mandibular fourth molars, premolars, and, less commonly, the canines and lateral incisors. Moreover, supernumerary teeth have been reported in areas of the oral cavity other than the alveolar ridges, such as the maxillary sinus, nasal cavity, soft palate, between the orbit and brain, and the sphenomaxillary fissure [2].

Supernumerary teeth are categorized based on their form and location. They are typically classified as either supplemental or rudimentary. Supplemental teeth exhibit a morphology and size comparable to that of normal teeth, whereas rudimentary types are typically smaller and display abnormal shapes. Rudimentary supernumerary teeth can be further categorized into subtypes, such as conical (peg-shaped), tuberculate (barrel-shaped with one or more cusps, usually found in the anterior region), and molariform or premolar-like forms [3]. Mesiodentes are most commonly found in the maxillary incisor region. The eruption process of conical mesiodens is spontaneous, whereas tuberculate-shaped supernumeraries are less frequent and usually remain unerupted. A distomolar, also known as distodens, refers to a fourth molar. In contrast, a paramolar is defined as a supernumerary tooth located in the posterior region, positioned either buccally or lingually to a molar [2]. Overall, the presence of a supernumerary tooth may result in the failed or delayed eruption of a permanent tooth, as well as contribute to the development of a large diastema or incisor malalignment as potential clinical implications [3].

Supernumerary teeth occur either sporadically or as a result of hereditary factors. Specifically, cleidocranial dysplasia, a genetic disorder linked to the presence of multiple supernumerary teeth, follows an autosomal dominant inheritance pattern and is characterized by genetic mutations of high prevalence [1].

The occurrence of supernumerary teeth varies among ethnic groups, with Caucasians showing a prevalence of 0.1% to 3.8%, while Asians exhibit a slightly higher percentage. The frequency in black Americans is up to nine times greater compared to white Americans. There is a male predilection of two to one compared to females in terms of gender. In terms of hyperdontia types, single supernumerary teeth are observed in approximately 76% to 86% of cases and are most commonly found in the permanent dentition. Most supernumerary teeth are located in the maxilla, especially in the anterior area. The presence of two supernumerary teeth occurs in 12% to 23% of cases, while occurrences of three or more are rare, accounting for less than 1% [3]. Several studies have investigated the prevalence of supernumerary and impacted teeth in the Greek population. Fardi et al. found a prevalence of 1.8% in Northern Greece, aligning with international reports. Their findings highlighted mesiodens as the most common supernumerary tooth, contributing to the understanding of the distribution of dental anomalies across different regions of Greece [4]. Recently, Lykousis et al. evaluated a Greek pediatric population, identifying the prevalence of 11.6%, with a male predilection [5]. Although the prevalence of supernumerary teeth has been reported in general populations, few studies have explicitly focused on growing individuals from geographically isolated areas.

Compared to panoramic radiographs, upper occlusal or periapical radiographs offer improved diagnostic accuracy for identifying anterior supernumerary teeth, due to the reduced image quality of panoramic radiographs in that region. While periapical radiographs combined with the parallax technique are useful for locating anterior supernumerary teeth, vertex occlusal radiographs provide superior accuracy in evaluating their anteroposterior position. Additionally, digital imaging with cone-beam computed tomography (CBCT) enables high-definition, three-dimensional imaging while exposing the patient to less radiation compared to conventional computed tomography [1].

The presence of supernumerary teeth in the premaxilla is usually associated with various clinical manifestations and, quite often, with impaction of the central incisors [6]. To prevent the risk of complications affecting adjacent teeth, such as eruption delays, resorption of roots, displacement from crowding, dilaceration, ectopic nasal eruption, malocclusion, and diastema formation, the timely extraction of the supernumerary tooth during the mixed dentition stage is recommended. These complications occur more frequently in the anterior region than in the posterior. In most cases, the permanent tooth erupts naturally following supernumerary extraction. However, if the apices of the permanent tooth are closed and it is still unerupted, surgical exposure combined with orthodontic treatment may be required to facilitate eruption [2]. Among the various types of supernumerary teeth, tuberculate mesiodentes are characterized by a higher incidence of eruption failure, which can lead to crowding, displacement, or rotation of adjacent teeth, often necessitating orthodontic intervention [7,8].

The objective of this study was to investigate the prevalence of supernumerary teeth among Caucasians from the Greek island of Lesvos. Located in the northeastern Aegean Sea, Lesvos is the third-largest Greek island and ranks eighth among all Mediterranean islands. Since Greece encompasses both mainland areas and a substantial number of islands, the authors found it particularly interesting to analyze the prevalence of supernumerary teeth in the island population of Lesvos as part of their examination of dental anomalies.

## 2. Materials and Methods

This study followed a 10-year retrospective cross-sectional design and adhered to all applicable guidelines and regulations. It was conducted in accordance with the Declaration of Helsinki and was approved by the Ethical Committee at Dr. M. Katanakis’ Clinic in Mytilini, Lesvos, on 12 October 2022. We utilized anonymized archived data from existing diagnostic records, which encompassed panoramic radiographs in combination with available periapical radiographs, before any interventions were made.

**Figure 1:**
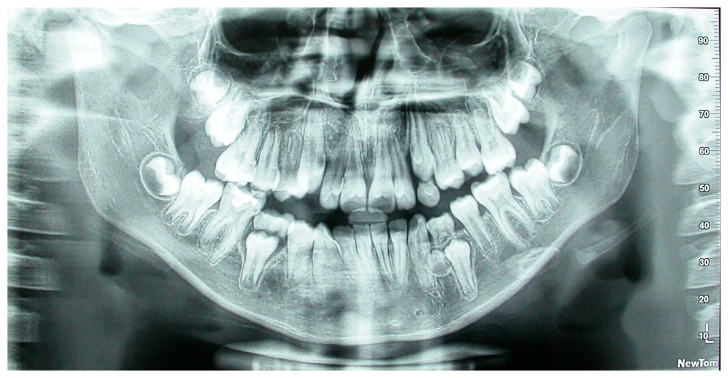
Panoramic radiograph of a patient with supernumerary mandibular left right premolar.

**Figure 2:**
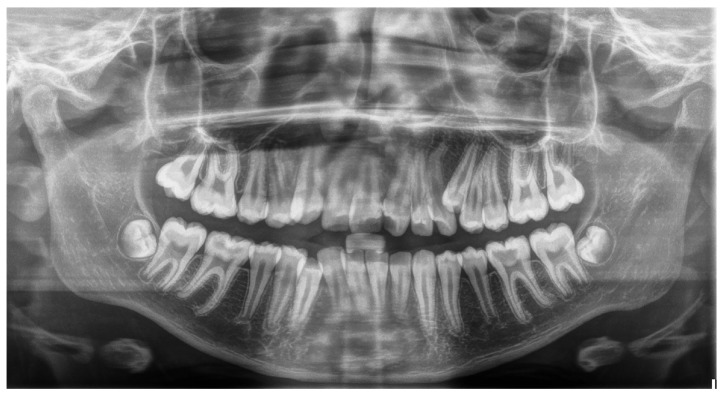
Panoramic radiograph of a patient with supernumerary maxillary left premolar.

Our inclusion criteria were the following:

1. Participants had to be children and adolescents aged 9 to 16;
2. Both their parents should be of native Greek descent from Lesvos;
3. Clear, high-quality panoramic X-rays (Figure 1 and Figure 2) should be available.

Τo confirm that no additional supernumerary teeth developed later, post-orthodontic treatment radiographs were re-evaluated. Since no newly formed supernumerary teeth were identified in our sample, only the initial records were considered for the purpose of this study. The participants were selected using convenience sampling; nevertheless, its demographic characteristics aligned closely with those of the island’s pediatric population, enabling reasonably representative prevalence estimates.

Meanwhile, the exclusion criteria were the following:

1. Individuals were excluded if their parents were not of Greek origin or originated from different regions of Greece.
2. Children with syndromes or conditions associated with hyperdontia (including ectodermal dysplasia, cleft lip, and palate) were excluded.

A total of 88 records were excluded on this basis.

According to the previously described criteria, a convenience sample of 621 children (258 males and 363 females) was recruited from orthodontic clinics and general/pediatric dental practices for inclusion in the study. These participants were drawn from a larger group of 709 children. Sample selection was based on the availability of diagnostic radiographs meeting the inclusion criteria. Patients were included during the study period, if their records satisfied the eligibility requirements. The focus on growing patients aged 9 to 16 years was intentional, as this period allows for the dentification of both early and late developing supernumerary teeth that may affect the developing occlusion and facial growth. The prevalence of supernumerary teeth was assessed. All radiographs were examined on a dental X-ray viewer by two authors (N.K. and M.A.M.), whose academic backgrounds and university education qualified them to evaluate panoramic radiographs. A tooth was classified as supernumerary if the normally expected number of teeth per type was exceeded. Descriptive statistics were calculated using Microsoft Excel (version 16.95.1, Microsoft Corporation, Redmond, WA, USA). Given the limited number of positive cases (n = 15), only descriptive statistics were applied.

## 3. Results

The mean age of the 621 children in the study group was 12.5 years. The prevalence of supernumerary teeth was 2.4% (6 females and 9 males), which gave a total of 15 patients with a supernumerary tooth (Table 1).

The maxillary region exhibited the highest occurrence of supernumerary teeth. Most supernumerary teeth were found in the maxilla (Table 2). The most common supernumerary teeth were mesiodentes (n = 8), followed by supernumerary lateral incisors (n = 3), paramolars (n = 2), and supernumerary central incisors (n = 1) (Table 3).

## 4. Discussion

In the present study, the prevalence of supernumerary teeth was found to be 1.9% in the maxilla and 0.5% in the mandible. The most common types, in descending order of frequency, were mesiodens, supernumerary lateral incisors, paramolars, and supernumerary central incisors. As the sample consisted of otherwise healthy individuals, orthodontists should always maintain a high level of vigilance for the detection of hyperdontia.

Conditions and syndromes associated with supernumerary teeth include Nance Horan syndrome, amelogenesis imperfecta, Marfan syndrome, Hallerman–Streiff syndrome, Crouzon syndrome, Franceschetti syndrome, Rubinstein–Taybi syndrome, Down syndrome, Noonan syndrome, Ellis–Van Creveld syndrome, Zimmermann–Laband syndrome, Fabry disease, Ehlers–Danlos syndromes types III and IV, Robinow syndrome, Rothmund–Thomson syndrome, incontinentia pigmenti, Gardner syndrome, tricho-rhino-phalangeal syndrome, anophthalmia syndrome, cleidocranial dysplasia, craniosynostosis, cleft lip and palate, and orofaciodigital syndrome type I [9,10,11]. Overall, the presence of multiple supernumerary teeth is uncommon and generally associated with syndromic conditions [12,13].

Hyperdontia leads to various complications that may impact both aesthetics and oral health, such as crowding, root resorption, midline diastema, formation of cystic lesions, ectopic eruption, displacement of the crowns of adjacent teeth, intraoral infections, and delayed or inhibited eruption [9,10,12,13,14,15,16]. Nonetheless, in certain situations, supernumerary teeth might not interfere with occlusion or cause other issues [16]. Early detection and effective management are vital for minimizing or preventing the adverse effects discussed above. Clinical evaluation is the primary and most critical step for identifying impacted teeth [17,18]. This typically includes visual inspection and palpation, while radiographic examination may utilize various imaging techniques [19]. Precise localization of supernumerary teeth is crucial for accurate diagnosis and effective treatment planning, particularly in cases that necessitate surgical intervention [20].

The typical treatment strategy includes the removal of supernumerary teeth [12,21]. Some experts propose that in cases where a supernumerary tooth has emerged in a suitable position and does not compromise occlusion, it may be retained and periodically monitored, provided it poses no risk to adjacent structures. However, in cases where there is insufficient space, early extraction is advised to avoid crowding and occlusal complications [12,22]. For impacted supernumerary teeth, prompt extraction may be recommended before any scheduled orthodontic procedures to prevent complications and facilitate optimal dental alignment [13,23]. Nonetheless, some specialists advocate delaying extraction until the roots of the supernumerary tooth have fully developed and/or the permanent teeth have completely erupted to reduce the risk of unintentional harm to adjacent teeth or nearby anatomical structures [12,13,24].

The 2.4% prevalence of supernumerary teeth observed in this study closely matches findings from Syriac et al. [25], Demiriz et al. [26], Dobles and Meza [27], and the review by Mallineni et al. [28]. Although our data is limited, our results indicate a slight male predilection, which aligns with earlier research. Singh et al. [29] evaluated 3000 children and reported a male-to-female ratio of 2.29:1. Arikan et al. [30] examined 7551 patients and identified a male-to-female ratio of 1.84:1, with 48 males and 26 females presenting with supernumerary teeth. Jain et al. [31] studied 4000 Indian subjects and found a prevalence of hyperdontia of 1.2% in males compared to 1.05% in females. The same trend has been consistently reported by Anegundi et al. [32], McBeain and Miloro [33], Irish [34], and Mahabob et al. (2012) [35]. Overall, the condition appears to be more prevalent in males.

Regarding the prevalence of supernumerary teeth among various ethnic groups, a study by Sella Tunis et al. [36] evaluated the pretreatment records of 2897 Caucasian patients and observed that maxillary supernumerary teeth were most prevalent, particularly affecting the incisors (59.38%). The mesiodens was identified as the most common type of supernumerary tooth (37.5%). These findings are consistent with our results regarding both the most commonly affected area and the increased occurrence of supernumerary teeth. Furthermore, research by Eshgian et al. [37] examined dental anomalies across diverse ethnic groups, including Hispanics, African Americans, Asians, and Caucasians, revealing that hyperdontia was most frequently observed in Hispanic patients at a rate of 39.24%. Moreover, Bello et al. [38] assessed 1837 radiographs from two dental practices in Nigeria. They reported a low prevalence of hyperdontia among different African ethnic groups, with rates of 1.47% for unilateral and 0.27% for bilateral hyperdontia. Furthermore, a study by Shen et al. [39], reviewing panoramic radiographs and medical records of 5015 Asian patients, found a prevalence rate of 4.03%, with a notable predilection for the maxilla.

According to the current literature, the most frequent type of supernumerary tooth is mesiodens, followed by supernumerary premolars, laterals, distomolars, and paramolars [40]. Similarly, a study by Çelikoglu et al. [41] also identified mesiodens as the most common type, followed by premolars, laterals, distomolars, paramolars, and canines. The reported sequences of prevalence for supernumerary teeth other than mesiodentes diverge from our research, which reported a descending prevalence of supernumerary laterals, paramolars, and central incisors.

Also, “non-syndromic late-developing supernumerary teeth” have been observed and mentioned in the relevant bibliography. This term refers to supernumerary teeth that emerge later than the expected age of the associated teeth, without being linked to any syndromes. Late-developing supernumerary teeth can occur as single or multiple [42,43,44] and may be unilateral or bilateral [45,46], affecting the mandible, maxilla, or both arches [47,48,49]. These teeth can be situated in multiple regions of dental arches, such as the canine, premolar, and molar regions [50,51].

In the present study, we focused on the growing population (ages 9–16) because this age range allows for early identification of supernumerary teeth and enables timely orthodontic or surgical intervention. Additionally, upon completion of treatment for the included patients, we re-evaluated their final records to identify any potentially newly developed supernumerary teeth. However, none was found. Moreover, this age window aligns with the typical developmental stages of permanent dentition. Given Lesvos’ relatively homogenous population, this study may provide insights into region-specific patterns of dental anomalies, possibly reflecting underlying genetic predispositions or environmental influences unique to island populations.

To the best of our knowledge, this study is among the few to investigatehe few to investigate the prevalence of supernumerary teeth in Greece, particularly within an island population. Most prior Greek research has employed CBCT imaging for detection [4,5]. Fardi et al. found a prevalence of 1.8% [4], whereas Lykousis et al. reported a higher rate of 11.6% [5]. In the wider Balkan region, specifically Bosnia and Herzegovina, Hadziabdic et al. noted a prevalence of 0.98%, with mesiodens being the most common type [52]. These previous and current findings offer important data for mapping dental anomalies geographically and could aid future research into the genetic and environmental factors affecting dental development in isolated populations.

The present study builds upon the existing epidemiological knowledge of dental anomalies in this geographic region, extending our previous research from two years ago that focused on the prevalence of congenitally missing permanent teeth on Lesvos Island [53]. The research adhered to a standardized protocol developed at the outset, with all investigators trained and calibrated to accurately identify supernumerary teeth, thereby ensuring consistent application of the inclusion criteria. A notable limitation of the study, however, was its reliance on a convenience sample, as it was not feasible to reach the entire population of such a large island.

## 5. Conclusions

The prevalence of supernumerary teeth was 2.4%, with the upper mesiodens being the most commonly observed tooth. A similar prevalence was noted for paramolars and central incisors. The maxilla had the highest number of supernumerary teeth.

## Figures and Tables

**Table 1:** Distribution of individuals with supernumerary teeth by gender.

	Number	Percentage
Females	6	1%
Males	9	1.4%
Total	15	2.4%

**Table 2:** Distribution of supernumerary teeth by jaw.

Prevalence of Supernumerary Teeth by Arch				
	Males	Females	Total	Percentage
Maxilla	7	5	12	1.9%
Mandible	2	1	3	0.5%

**Table 3:** Distribution of supernumerary teeth by tooth type and gender.

	Males	Females	Total	Percentage
Mesiodentes	5	3	8	1.3%
Supernumerary Lateral incisors	2	1	3	0.5%
Paramolars	1	1	2	0.3%
Supernumerary Central incisors	1	1	2	0.3%

## Data Availability

The supporting data reported can be provided upon request to the corresponding author.
